# Systematic Review and Meta-Analysis of Risk Factors Associated with Postoperative Stress Hyperglycemia in Patients without Diabetes Following Cardiac Surgery

**DOI:** 10.31083/RCM25485

**Published:** 2025-01-21

**Authors:** Mengli Zhang, Ziyi Zhang, Ningning Zhu, Lulu Wang, Hui Huang, Yike Wang, Fang Xue

**Affiliations:** ^1^School of Nursing, Bengbu Medical University, 233030 Bengbu, Anhui, China; ^2^Department of Cardiac Surgery, The First Affiliated Hospital of Bengbu Medical University, 233000 Bengbu, Anhui, China; ^3^Clinical Medical School, Zhengzhou University, 450001 Zhengzhou, Henan, China

**Keywords:** cardiac surgery, stress-induced hyperglycemia, risk factors, systematic review and meta-analysis

## Abstract

**Background:**

To systematically evaluate risk factors for stress-induced hyperglycemia in patients without diabetes after cardiac surgery.

**Methods:**

Databases including CNKI, WanFang data, VIP, SinoMed, PubMed, Web of Science, Embase, and the Cochrane Library were searched using computer retrieval. The data were subjected to an in-depth meta-analysis using RevMan 5.4 and Stata 15.0 software.

**Results:**

This study involved 11,645 postoperative cardiac surgery patients, including 8 case-control studies and 3 cohort studies, over which 18 risk factors were identified. The results of the meta-analysis indicated that statistically significant risk factors included age >65 years [odds ratios (OR) (95% CI ) = 3.47 (2.61–4.32)], female gender [OR (95%) = 1.54 (1.34–1.76)], combined heart valve and coronary artery bypass surgery [OR (95%) = 1.82 (1.23–2.70)], ejection fraction <40% [OR (95%) = 1.38 (1.17–1.63)], history of heart surgery [OR (95%) = 1.30 (1.06–1.59)], myocardial infarction [OR (95%) = 1.17 (1.05–1.31)], hyperlipidemia [OR (95%) = 0.76 (0.67–0.86)], hypertension [OR (95%) = 1.12 (1.03–1.22)], anticoagulant medication [OR (95%) = 0.77 (0.65–0.90)], cardiopulmonary bypass time >2 hours [OR (95%) = 20.26 (17.03–23.48)] and history of cardiopulmonary bypass [OR (95%) = 1.24 (1.09–1.41)].

**Conclusions:**

Current evidence suggests that there are key risk factors for postoperative stress hyperglycemia in patients without diabetes who have undergone cardiac surgery. These factors can help identify patients at a high risk of perioperative stress hyperglycemia during cardiac surgery. This evidence provides a basis for healthcare professionals to develop predictive management strategies for perioperative stress hyperglycemia in patients without diabetes. However, more high-quality studies are required to address the limitations of the current research.

**The PROSPERO registration:**

CRD42024479215, https://www.crd.york.ac.uk/PROSPERO/display_record.php?RecordID=479215.

## 1. Introduction

Stress hyperglycemia (SHG) refers to a temporary increase in blood glucose levels under high-stress conditions, such as major trauma, severe infection, or cardiovascular incidents, in patients with no prior history of diabetes [1, 2]. According to the American Diabetes Association (ADA) consensus, SHG is defined as a fasting blood glucose level ≥7.0 mmol/L or random blood glucose values ≥11.1 mmol/L on two or more occasions [3]. The common complications of cardiac surgery include SHG [2], pulmonary infection [4], anemia [5], ischemic cerebrovascular disease [6], postoperative neurocognitive dysfunction [7], arrhythmias [8], and seizures [9]. The incidence of SHG post-cardiac surgery is approximately 75%. Hyperglycemia is prevalent among postoperative cardiac patients, with early postoperative peak blood glucose levels persisting for an extended period, adversely affecting prognosis. Furthermore, the occurrence of SHG can lead to osmotic diuresis, causing disturbances in water, electrolytes, and acid-base balance, thereby posing a threat to the health of cardiac surgery patients [10]. Although studies [11, 12] have investigated risk factors for SHG in patients without diabetes after cardiac surgery, the inconsistency in the factors included in these studies necessitates a comprehensive analysis to minimize potential biases from different research methodologies and measurement tools. This study aimed to investigate risk factors for SHG in patients without diabetes after cardiac surgery through a literature review and meta-analysis. Currently, research on SHG in patients without diabetes undergoing cardiac surgery is largely limited to analyzing the influencing factors. The innovation of this study lies in its focus on patients without diabetes, an area often overlooked in research, as most studies primarily address postoperative glycemic management in diabetic patients. The risk and impact of SHG in patients without diabetes following surgery are frequently underestimated. Research [13] has shown that perioperative stress hyperglycemia in cardiac surgery not only increases the incidence of postoperative complications and adverse cardiac events but also prolongs intensive care unit (ICU) stays, extends total hospitalization time, and increases postoperative mortality rates. However, no comprehensive systematic review or meta-analysis has specifically addressed SHG in patients without diabetes after cardiac surgery. This study provides a more comprehensive exploration through a systematic review and meta-analysis, incorporating rigorous evidence-based methods. By searching relevant databases and conducting a strict quality assessment of the literature, this study synthesized and interpreted the findings. It not only offers guidance for clinical practice but also provides evidence to support early perioperative interventions and supportive treatments and enables clinicians to more accurately identify high-risk patients during perioperative evaluations in cardiac surgery. By uncovering the complexity of various influencing factors, this systematic review and meta-analysis emphasized the importance of multidisciplinary collaboration in postoperative management. These research findings encourage cardiac surgeons, endocrinologists, and anesthesiologists to work together to create and implement perioperative management plans, ultimately improving overall patient outcomes.

## 2. Literature Review

### 2.1 Literature Search Strategy

A comprehensive literature search was conducted using several databases, including CNKI, Wanfang Data, VIP, SinoMed, PubMed, Web of Science, Embase, and the Cochrane Library. The search period was extended to December 1, 2023. The included literature consisted of works in Chinese, English, and other languages, as well as grey literature, employing a combination of subject and free terms. The references of the included studies were also traced. The Chinese search terms included “off-pump coronary artery bypass”, “cardiac surgery”, “stress hyperglycemia”, “hyperglycemia”, “stress-induced hyperglycemia”, “risk factors”, and “influencing factors”. The English search terms included (“cardiac operation” OR “cardiac surgical procedures”) OR (“coronary artery bypass, off-pump”) AND (“hyperglycemia” OR “irritable hyperglycemia” OR “stress hyperglycemia”).

### 2.2 Inclusion and Exclusion Criteria

#### 2.2.1 Inclusion Criteria

2.2.1.1 Diagnosis and Measurement of Stress Hyperglycemia

The review Stress Hyperglycemia, published in the prestigious journal The Lancet [3], mentioned that SHG typically refers to transient hyperglycemia occurring during illness, usually in patients without a prior history of diabetes. The ADA defines hospital-related hyperglycemia as fasting blood glucose >7 mmol/L or random blood glucose >11.1 mmol/L in patients without a known history of diabetes. Excluding patients with glycosylated hemoglobin (HbA1c) levels ≥6.5%. HbA1c levels can be used to assess stress-induced hyperglycemia. HbA1c reflects the average blood glucose level over the past 2–3 months; Individuals with a history of diabetes or impaired glucose regulation will have elevated HbA1c levels, while those with stress hyperglycemia, due to its short-term nature, typically have normal HbA1c levels [14]. Such as drug-induced hyperglycemia, hyperthyroidism, transient hyperglycemia due to acute pancreatitis, and endocrine tumors should be excluded.

2.2.1.2 Study Types

Case-control studies and Cohort studies.

2.2.1.3 Postoperative Monitoring Timing

The first 24–48 hours [15] after surgery are a high-risk period for stress-induced hyperglycemia. Therefore, frequent blood glucose monitoring is necessary, especially when insulin or other interventions are being used.

2.2.1.4 Monitoring Methods

① Capillary Blood Glucose Monitoring: Blood is drawn from the fingertip and tested immediately using a portable glucometer. This method is convenient and quick, but may be affected by local circulation conditions. ② Venous Plasma Glucose Measurement: Blood is drawn from a vein, and the glucose concentration in the plasma is measured in a laboratory. This method is more accurate than capillary blood glucose testing but slower, and is typically used for diagnosis and precise monitoring. ③ Continuous Glucose Monitoring (CGM): A subcutaneous sensor is used to monitor blood glucose levels in real time, providing continuous tracking of glucose fluctuations. This is particularly useful for postoperative monitoring of stress-induced hyperglycemia, especially in critically ill patients who require close observation.

2.2.1.5 Study Subjects

Patients without diabetes who underwent cardiac surgery, regardless of race, nationality, or disease duration.

2.2.1.6 Exposure Factors

Exposure factors included general risk factors potentially associated with the patient during the perioperative period, preoperative comorbidities, perioperative medications, and certain intraoperative risks related to extracorporeal circulation.

2.2.1.7 Outcome Measure

The occurrence of SHG after cardiac surgery.

#### 2.2.2 Exclusion Criteria

① Newcastle-Ottawa Scale (NOS) Score Below 6 [16]. ② Studies that were duplicates or suspected of being duplicate reports were excluded. ③ Studies with incomplete, insufficient, or erroneous data were excluded. ④ Exclusion criteria included the following: impaired fasting glucose (IFG) patients (fasting blood glucose 5.6–6.9 mmol/L, with a 2 hour postprandial blood glucose <7.8 mmol/L), impaired glucose tolerance (IGT) patients (fasting blood glucose <5.6 mmol/L, with a 2 hour postprandial blood glucose between 7.8–11.0 mmol/L) and patients with combined IFG and IGT (fasting blood glucose 5.6–6.9 mmol/L, with 2-hour postprandial blood glucose between 7.8–11.0 mmol/L) [14].

### 2.3 Introduction to the NOS for Assessing Study Quality 

The NOS [16] is a tool specifically designed to assess the quality of non-randomized studies, including case-control and cohort studies. Given the potential biases and confounding factors inherent in non-randomized studies, it is essential to evaluate their quality. The NOS helps researchers to systematically and quantitatively assess whether the design, execution, and interpretation of results in these studies are scientifically sound and valid. The NOS evaluates studies across three key domains: ① Selection: Assesses whether the selection of cases or controls in the study is appropriate. ② Comparability: Evaluates how well the study controls confounding factors between the study and control groups to ensure that the results are comparable. ③ Outcome/Exposure: Examines whether the measurement of exposure or outcomes is accurate, consistently applied across study and control groups, and considers issues such as non-response rates. The Selection domain is scored out of 4 points, Comparability out of 2 points, and Outcome/Exposure out of 3 points, with a total score ranging from 0 to 9. Higher scores indicate better study quality and a lower risk of bias.

### 2.4 Quality Assessment of Selected Studies 

A NOS score of ≥6 indicates a high-quality process of evaluating the quality of the literature [16], if there is a disagreement between the two researchers’ conclusions, a third-party expert may be consulted for discussion and adjudication, or multiple rounds of discussion may be conducted until consensus is reached. If necessary, the original authors were contacted to obtain information relevant to quality assessment.

### 2.5 Literature Screening and Data Extraction 

Two researchers independently performed literature screening and data collection. After completing independent data extraction, the two sets of extracted data were compared with particular attention to key variables (such as sample size and region). For any discrepancies, the original literature was reviewed to verify which set of data was correct; if discrepancies arose, discussions were held to reach a consensus, and third-party consultation was sought if necessary. The initial screening involved reviewing titles to exclude irrelevant articles, followed by abstract and full-text reviews to determine inclusion. When required, the original authors were contacted for additional data. The study primarily included basic information about the research participants, such as the first author, region, and publication date, as well as demographic data, including sample size and age. It also included the number of patients and main factors for bias risk assessment. The outcome measures and related data were reviewed independently by two researchers. If disagreements occurred, a third-party expert was consulted, or multiple discussions were held until a consensus was reached.

### 2.6 Statistical Methods Application 

Data were analyzed that the Revman 5.4 software (Cochrane Collaboration, Oxford, Oxon, UK), developed by the UK Cochrane Collaboration, and the Stata 15.0 software (Statacorp, college station, TX, USA), created by the US Computer Resource Center. Count data are expressed as odds ratios (OR) and continuous data as mean differences (MD), with each effect presented as a point estimate and a 95% confidence interval (CI). Heterogeneity was assessed using Chi-squared (χ^2^) test with α = 0.1 significance level, complemented by I^2^ values for quantitative heterogeneity assessment. When no significant differences were found between groups (I^2^ < 50%, *p* > 0.1), a fixed-effects model was used for the meta-analysis. Conversely, when significant differences existed (I^2^ ≥ 50%, *p* < 0.1), a random-effects model was applied. Studies showing significant clinical heterogeneity were further analyzed using subgroup, sensitivity, and descriptive methods. Meta-analyses were conducted at a significance level of α = 0.05.

### 2.7 Database Registration 

This study has been registered in the National Institute for Health Research (NIHR) PROSPERO database, registration number CRD42024479215 (https://www.crd.york.ac.uk/PROSPERO/display_record.php?RecordID=479215).

## 3. Results

### 3.1 Literature Search and Screening Results

**Fig. 1.**
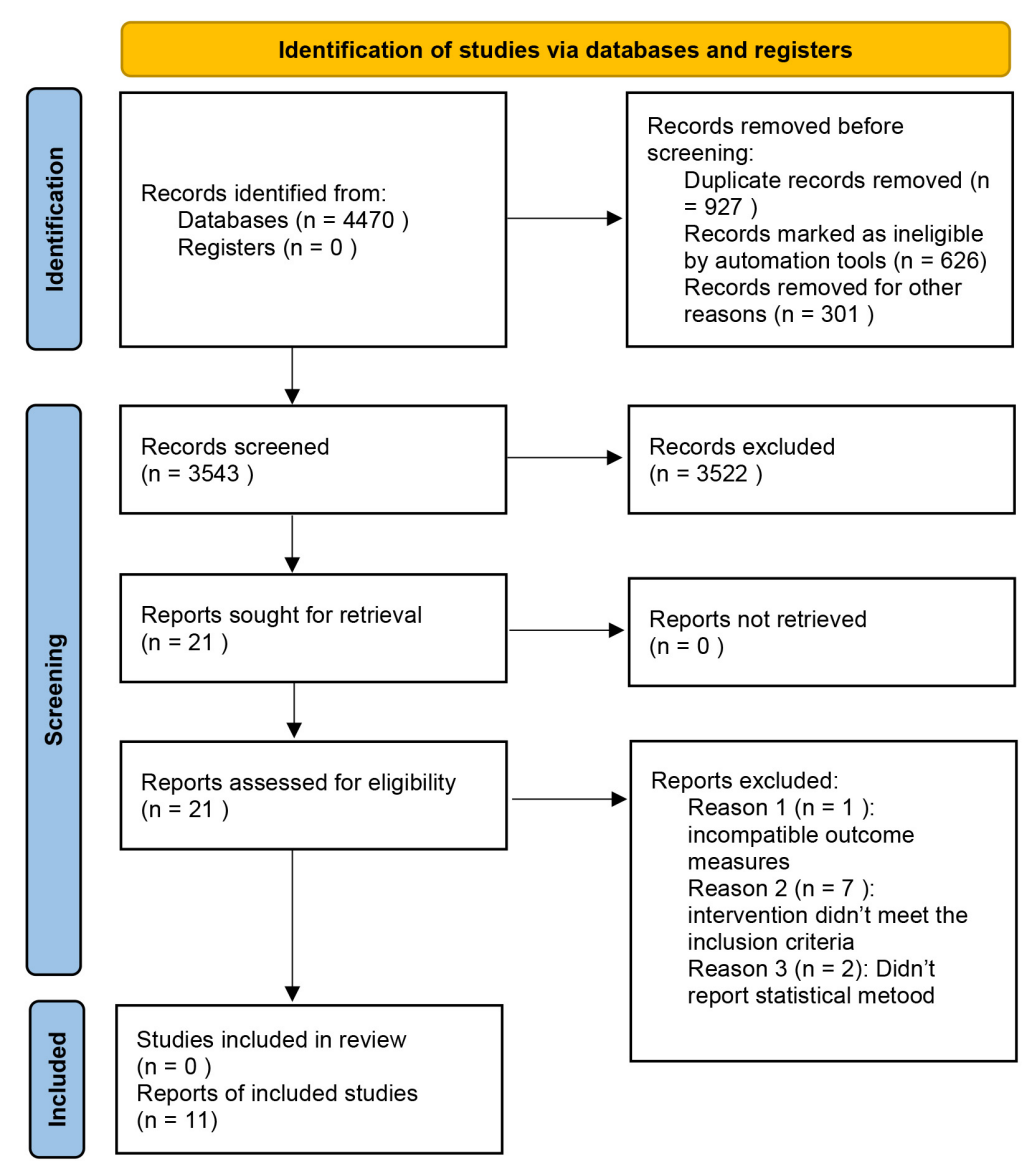
**Literature screening flow chart**.

A total of 4470 relevant articles were retrieved from the CNKI (n = 141), VIP (n = 2), WanFang Data (n = 144), SinoMed (n = 118), PubMed (n = 1724), Web of Science (n = 1013), Embase (n = 779), and Cochrane Library (n = 549) databases. This study included 405 articles in Chinese and 4065 articles in English. The articles were imported into EndNote and duplicates were removed. After the initial screening of titles and abstracts, 3543 articles were selected for further review. Ultimately, 11 articles were included in this study, comprising eight case-control studies [7, 11, 13, 17, 18, 19, 20, 21] and three cohort studies [12, 22, 23]. These studies involved 11,645 patients who underwent cardiac surgery-related procedures. The detailed literature screening process is illustrated in Fig. 1.

### 3.2 Basic Characteristics of Included Study

A total of 4470 relevant articles were retrieved from the following databases: CNKI (n = 141), VIP (n = 2), WanFang Data (n = 144), SinoMed (n = 118), PubMed (n = 1724), Web of Science (n = 1013), Embase (n = 779), and Cochrane Library (n = 549). This study included 405 articles in Chinese and 4065 articles in English. The articles were imported into EndNote and duplicates were removed. After the initial screening of titles and abstracts, 3543 articles were selected for further review. Ultimately, 11 articles were included in this study, comprising eight case-control studies [7, 11, 13, 17, 18, 19, 20, 21] and three cohort studies [12, 22, 23]. These studies involved 11,645 patients who underwent cardiac surgery-related procedures. The detailed literature screening process is illustrated in Table 1 (Ref. [7, 11, 12, 13, 17, 18, 19, 20, 21, 22, 23]), Table 2 (Ref. [12, 22, 23]), Table 3 (Ref. [7, 11, 13, 17, 18, 19, 20, 21]).

**Table 1.** **General information about the included literature**.

Include in references	Country	Study type	Age (years)	Researching spells	Sample size	Type of operation	Number of cases	Incidence	Risk factors
Amit A. Prasad *et al*. 2007 [7]	USA	case control study	66–70	2004–2006	162	Cardiac surgery	35	21.6%	①②⑤⑧⑩⑪⑫⑬⑭⑮⑰
Vikaesh Moorthy *et al*. 2019 [11]	Singapore	case control study	46.5–69.7	2008–2010	1602	CABG	898	56.0%	②⑤⑩⑫⑭⑮⑰⑱
Rajesh Garg *et al*. 2013 [12]	USA	cohort study	50.9–78.3	2004–2009	3658	Valve surgery and CABG	1453	40.0%	①②③④⑤⑥⑦⑧⑨⑩⑰
Russell E. Anderson *et al*. 2005 [22]	Sweden	cohort study	64–69	2004	45	CABG	23	51.1%	①②⑤⑦⑧⑩⑪⑯
Roma Y. Gianchandani *et al*. 2015 [23]	USA	case control study	55–67	2015	61	Valve surgery and CABG	49	80.3%	-
Jiang Jiduan 2016 [17]	China	case control study	46.3–63.9	2014–2015	69	Valve surgery	27	39.1%	②⑰
Xiaojue Li *et al*. 2023 [13]	China	cohort study	54–67	2011–2014	5450	CABG	4342	79.7%	②③④⑥⑦⑧⑨⑩⑫⑬⑭⑮⑯⑱
Utkan Sevuk *et al*. 2014 [18]	Turkey	case control study	50.2–72	2011–2014	200	CABG	100	50.0%	②③
Yubin Chen *et al*. 2023 [19]	China	case control study	39.4–64.3	2016–2020	203	Type A aortic dissection	86	42.4%	②③⑤⑩
Shen Minwei *et al*. 2019 [20]	China	case control study	36–58	2016–2017	100	Type A aortic dissection	31	31.0%	②
Zhang Yan *et al*. 2010 [21]	China	case control study	25–75	2006–2008	95	Valve surgery and CABG	41	43.1%	-

① age >65 years old; ② female; ③ smoking history; ④ heart valve combined bypass surgery; ⑤ history of cardiac surgery; ⑥ cerebrovascular disease history; ⑦ myocardial infarction; ⑧ hyperlipidemia; ⑨ peripheral vascular disease; ⑩ hypertension; ⑪ unstable angina; ⑫ anticoagulant drug; ⑬ lipid-regulating drugs; ⑭ β-blocker; ⑮ angiotensin converting enzyme inhibitors; ⑯ extracorporeal circulation; ⑰ the duration of cardiopulmonary bypass >2 H; ⑱ ejection fraction <40%. CABG, coronary artery bypass grafting.

**Table 2.** **Evaluation of literature quality in cohort studies**.

Incorporate into study	Study population selection				Comparability	Ending			Total points
	(4 points)				(2 points)	(3 points)			
	Exposure group representativeness	Non-exposed group selection	Identification of exposure groups	Outcome events before the study begins	Comparability of exposed and unexposed groups	Outcome event evaluation	Adequacy of follow-up	Integrity of follow-up	
Rajesh Garg *et al*. 2013 [12]	1	1	1	0	2	1	0	1	7
Russell E. Anderson *et al*. 2005 [22]	1	1	1	1	2	1	1	0	8
Roma Y. Gianchandani *et al*. 2015 [23]	1	0	1	1	2	1	1	0	7

**Table 3.** **Evaluation of literature quality in case-control studies**.

Incorporate into study	Study population selection				Comparability	Ending			Total points
	(4 points)				(2 points)	(3 points)			
	Whether cases are properly defined and diagnosed	Case representation	Contrast selection	Definition of contrast	Comparability of cases and controls	Methods of investigation and assessment of exposure	Case and control investigation methods	Non response rate	
Amit A. Prasad *et al*. 2007 [7]	1	1	1	1	2	1	1	0	8
Vikaesh Moorthy *et al*. 2019 [11]	1	1	1	1	2	1	1	0	8
Xiaojue Li *et al*. 2023 [13]	1	1	1	0	2	1	0	0	6
Jiang Ji duan 2016 [17]	1	1	1	1	2	1	1	0	8
Utkan Sevuk *et al*. 2014 [18]	1	1	1	0	2	1	1	0	7
Yubin Chen *et al*. 2023 [19]	1	1	1	1	2	1	0	1	8
Shen Minwei *et al*. 2019 [20]	1	1	1	1	2	1	1	0	8
Zhang Yan *et al*. 2010 [21]	1	1	1	1	2	1	1	0	8

### 3.3 Meta-Analysis Results

#### 3.3.1 Factors for SHG in Patients without Diabetes after Cardiac Surgery

3.3.1.1 Age >65 and its Relationship with SHG in Patients without Diabetes after Cardiac Surgery

Three studies [7, 12, 22] reported on the relationship between age >65 and SHG in patients without diabetes after cardiac surgery. The heterogeneity test showed low heterogeneity among the studies (I^2^ = 0%, *p* < 0.00001). The pooled analysis using a fixed-effect model revealed that age >65 significantly impacts SHG in patients without diabetes patients after cardiac surgery, with a statistically significant difference [weighted mean difference, WMD = 3.47, 95% CI (2.61, 4.32), Z = 7.98, *p* < 0.00001].

3.3.1.2 Gender and its Relationship with SHG in Patients without Diabetes after Cardiac Surgery

Nine studies [7, 11, 12, 13, 17, 18, 19, 20, 22] reported on the relationship between gender and SHG in patients without diabetes after cardiac surgery. The heterogeneity test revealed high heterogeneity among the studies (I^2^ = 83%, *p* < 0.00001). Sensitivity analysis, performed by excluding studies individually, identified Rajesh Garg *et al*. [12] as the source of heterogeneity. After excluding this study, the heterogeneity decreased to 41%. The pooled analysis using a fixed-effect model showed a significant relationship between gender and SHG in patients without diabetes after cardiac surgery, with a statistically significant result [WMD = 1.54, 95% CI (1.34, 1.76), Z = 6.20, *p* < 0.00001].

3.3.1.3 Smoking and its Relationship with SHG in Patients without Diabetes after Cardiac Surgery

Four studies [12, 13, 18, 19] examined the relationship between smoking and SHG in patients without diabetes after cardiac surgery. The heterogeneity test showed low heterogeneity among the studies (I^2^ = 29%, *p* = 0.24). The pooled analysis using a fixed-effect model revealed that smoking had no significant impact on the occurrence of SHG in patients without diabetes after cardiac surgery, with no statistically significant difference [WMD = 0.97, 95% CI (0.89, 1.06), Z = 0.62, *p* = 0.53].

3.3.1.4 Combined Valve and Coronary Artery Bypass Surgery and its Relationship with SHG in Patients without Diabetes after Cardiac Surgery

Two studies [12, 13] reported on the relationship between combined valve and coronary artery bypass surgery and SHG in patients without diabetes after cardiac surgery. The heterogeneity test revealed high heterogeneity among the studies (I^2^ = 87%, *p* = 0.006). The pooled analysis using a random-effect model indicated that combined valve and bypass surgery significantly impacts SHG in patients without diabetes after cardiac surgery, with a statistically significant difference [WMD = 1.82, 95% CI (1.23, 2.70), Z = 3.01, *p* = 0.003].

3.3.1.5 Ejection Fraction <40% and its Relationship with SHG in Patients without Diabetes after Cardiac Surgery

Two studies [11, 13] investigated the relationship between ejection fraction <40% and SHG in patients without diabetes after cardiac surgery. The heterogeneity test showed low heterogeneity among the studies (I^2^ = 0%, *p* = 0.59). The pooled analysis using a fixed-effect model showed that an ejection fraction <40% significantly impacts SHG in patients without diabetes after cardiac surgery, with a statistically significant difference [WMD = 1.38, 95% CI (1.17, 1.63), Z = 3.75, *p* = 0.0002].

3.3.1.6 History of Cardiac Surgery and its Relationship with SHG in Patients without Diabetes after Cardiac Surgery

Four studies [7, 11, 12, 22] reported on the relationship between a history of cardiac surgery and SHG in patients without diabetes after cardiac surgery. The heterogeneity test showed low heterogeneity among the studies (I^2^ = 7%, *p* = 0.36). The pooled analysis using a fixed-effect model demonstrated that a history of cardiac surgery significantly impacts SHG in patients without diabetes after cardiac surgery, with a statistically significant difference [WMD = 1.30, 95% CI (1.06, 1.59), Z = 2.48, *p* = 0.01].

#### 3.3.2 Preoperative Comorbidity-Related Risk Factors for SHG in Patients without Diabetes after Cardiac Surgery

3.3.2.1 History of Cerebrovascular Disease and its Relationship with SHG in Patients without Diabetes after Cardiac Surgery

Two studies [12, 13] reported on the relationship between a history of cerebrovascular disease and SHG in patients without diabetes after cardiac surgery. The heterogeneity test showed low heterogeneity among the studies (I^2^ = 0%, *p* = 0.85). A pooled analysis using a fixed-effect model demonstrated that a preoperative history of cerebrovascular disease does not significantly impact SHG in patients without diabetes after cardiac surgery, with no statistically significant difference [WMD = 1.08, 95% CI (0.93, 1.26), Z = 0.98, *p* = 0.33].

3.3.2.2 Myocardial Infarction and its Relationship with SHG in Patients without Diabetes after Cardiac Surgery

Three studies [12, 13, 22] reported on the relationship between myocardial infarction and SHG in patients without diabetes after cardiac surgery. The heterogeneity test showed low heterogeneity among the studies (I^2^ = 12%, *p* = 0.32). The pooled analysis using a fixed-effect model revealed that a history of myocardial infarction significantly impacts SHG in patients without diabetes after cardiac surgery, with a statistically significant difference [WMD = 1.17, 95% CI (1.05, 1.31), Z = 2.76, *p* = 0.006].

3.3.2.3 Hyperlipidemia and its Relationship with SHG in Patients without Diabetes after Cardiac Surgery

Four studies [7, 12, 13, 22] examined the relationship between hyperlipidemia and SHG in patients without diabetes after cardiac surgery. The heterogeneity test revealed high heterogeneity among the studies (I^2^ = 90%, *p* < 0.00001). Sensitivity analysis, performed by excluding studies individually, identified Rajesh Garg *et al*. [12] as the source of heterogeneity. After excluding this study, heterogeneity decreased to 0%. Pooled analysis using a fixed-effects model revealed a significant relationship between hyperlipidemia and SHG in patients without diabetes after cardiac surgery, with a statistically significant difference [WMD = 0.76, 95% CI (0.67, 0.86), Z = 4.16, *p* < 0.0001].

3.3.2.4 History of Peripheral Vascular Disease and its Relationship with SHG in Patients without Diabetes after Cardiac Surgery

Two studies [12, 13] reported on the relationship between a history of peripheral vascular disease and SHG in patients without diabetes after cardiac surgery. The heterogeneity test showed high heterogeneity among the studies (I^2^ = 59%, *p* = 0.12). The pooled analysis using a random-effect model demonstrated that a preoperative history of peripheral vascular disease does not significantly impact SHG in patients without diabetes after cardiac surgery, with no statistically significant difference [WMD = 1.21, 95% CI (0.88, 1.66), Z = 1.18, *p* = 0.24].

3.3.2.5 Hypertension and its Relationship with SHG in Patients without Diabetes Patients after Cardiac Surgery

Six studies [7, 11, 12, 13, 19, 22] reported on the relationship between hypertension and SHG in patients without diabetes after cardiac surgery. The heterogeneity test showed low heterogeneity among the studies (I^2^ = 19%, *p* = 0.29). A pooled analysis using a fixed-effect model revealed that hypertension significantly impacts SHG in patients without diabetes after cardiac surgery, with a statistically significant difference [WMD = 1.12, 95% CI (1.03, 1.22), Z = 2.68, *p* = 0.007].

3.3.2.6 Unstable Angina and its Relationship with SHG in Patients without Diabetes after Cardiac Surgery

Two studies [7, 22] reported on the relationship between unstable angina and SHG in patients without diabetes after cardiac surgery. The heterogeneity test showed low heterogeneity among the studies (I^2^ = 0%, *p* = 0.80). The pooled analysis using a fixed-effect model demonstrated that unstable angina does not significantly impact SHG in patients without diabetes after cardiac surgery, with no statistically significant difference [WMD = 1.19, 95% CI (0.59, 2.41), Z = 0.48, *p* = 0.63].

#### 3.3.3 Medication-Related Risk Factors Before Surgery

3.3.3.1 Perioperative Use of Anticoagulants and its Relationship with SHG in Patients without Diabetes after Cardiac Surgery

Two studies [7, 13] reported a relationship between the perioperative use of anticoagulants and SHG in patients without diabetes after cardiac surgery. The heterogeneity test showed low heterogeneity among the studies (I^2^ = 0%, *p* = 0.60). The pooled analysis using a fixed-effect model revealed that perioperative use of anticoagulants significantly impacts SHG in patients without diabetes after cardiac surgery, with a statistically significant difference [WMD = 0.77, 95% CI (0.65, 0.90), Z = 3.30, *p* = 0.001].

3.3.3.2 Perioperative Use of Lipid-lowering Drugs and its Relationship with SHG in Patients without Diabetes after Cardiac Surgery

Two studies [7, 13] examined the relationship between the perioperative use of lipid-lowering drugs and SHG in non-diabetic patients after cardiac surgery. The heterogeneity test showed low heterogeneity among the studies (I^2^ = 0%, *p* = 0.91). The pooled analysis using a fixed-effect model demonstrated that perioperative use of lipid-lowering drugs does not significantly impact SHG in patients without diabetes after cardiac surgery, with no statistically significant difference [WMD = 0.88, 95% CI (0.73, 1.05), Z = 1.46, *p* = 0.14].

3.3.3.3 Perioperative Use of Beta-blockers and its Relationship with SHG in Patients without Diabetes after Cardiac Surgery

Two studies [7, 13] reported a relationship between the perioperative use of beta-blockers and SHG in patients without diabetes after cardiac surgery. The heterogeneity test showed low heterogeneity among the studies (I^2^ = 0%, *p* = 0.89). The pooled analysis using a fixed-effect model indicated that perioperative use of beta-blockers does not significantly impact SHG in patients without diabetes after cardiac surgery, with no statistically significant difference [WMD = 0.81, 95% CI (0.64, 1.03), Z = 1.70, *p* = 0.09].

3.3.3.4 Perioperative Use of Angiotensin-Converting Enzyme (ACE) Inhibitors and its Relationship with SHG in Patients without Diabetes after Cardiac Surgery

Three studies [7, 11, 13] reported on the relationship between perioperative use of ACE inhibitors and SHG in patients without diabetes after cardiac surgery. The heterogeneity test showed high heterogeneity among the studies (I^2^ = 89%, *p* = 0.0001). Sensitivity analysis identified Xiaojue Li *et al*. [13] as the source of heterogeneity. After excluding this study, heterogeneity decreased to 74%. The pooled analysis using a random-effect model showed that perioperative use of ACE inhibitors does not significantly impact SHG in patients without diabetes after cardiac surgery, with no statistically significant difference [WMD = 1.58, 95% CI (0.74, 3.38), Z = 1.19, *p* = 0.23].

#### 3.3.4 Intraoperative Extracorporeal Circulation-Related Risk Factors

3.3.4.1 Cardiopulmonary Bypass and its Relationship with SHG in Patients without Diabetes after Cardiac Surgery

Two studies [13, 22] reported the relationship between cardiopulmonary bypass (CPB) and SHG in patients without diabetes after cardiac surgery. The heterogeneity test indicated high heterogeneity among the studies (I^2^ = 0%, *p* = 0.76). Pooled analysis using a random-effects model showed that the use of CPB during cardiac surgery did not significantly affect the incidence of SHG in patients without diabetes postoperatively [WMD = 1.24, 95% CI (1.09, 1.41), Z = 3.24, *p* = 0.001].

3.3.4.2 CPB Duration >2 Hours and its Relationship with SHG in Patients without Diabetes after Cardiac Surgery

Three studies [7, 12, 17] investigated the relationship between CPB duration exceeding 2 hours and SHG in patients without diabetes after cardiac surgery. The heterogeneity test showed moderate heterogeneity among the studies (I^2^ = 47%, *p* = 0.17). The pooled analysis using a fixed-effects model demonstrated that the duration of cardiopulmonary bypass >2 H significantly impacted SHG in patients without diabetes postoperatively, with a statistically significant difference [WMD = 20.26, 95% CI (17.03, 23.48), Z = 12.31, *p* < 0.00001]. The detailed literature screening process is illustrated in Table 4 (Ref. [7, 11, 12, 13, 17, 18, 19, 20, 22]).

**Table 4.** **Summary map of risk of literature bias**.

Risk factor		Number of articles included	I^2^ (%)	*p*	OR	95% CI	Effect model
General risk factors for patients							
	Age >65 years	3 [7, 12, 22]	0%	<0.00001	3.47	2.61, 4.32	fixed effect model
	Female	8 [7, 11, 13, 17, 18, 19, 20, 22]	41%	<0.00001	1.54	1.34, 1.76	fixed effect model
	Smoking history	4 [12, 13, 18, 19]	29%	0.53	0.97	0.89, 1.06	fixed effect model
	Heart valve surgery combined with bypass surgery	2 [12, 13]	87%	0.003	1.82	1.23, 2.70	random effect model
	Ejection fraction <40%	2 [11, 13]	0%	0.0002	1.38	1.17, 1.63	fixed effect model
	History of cardiac surgery	4 [7, 11, 12, 22]	7%	0.01	1.30	1.06, 1.59	fixed effect model
Risk factors for preoperative comorbidities							
	Cerebrovascular disease history	2 [12, 13]	0%	0.33	1.08	0.93, 1.26	fixed effect model
	Myocardial infarction	3 [12, 13, 22]	12%	0.006	1.17	1.05, 1.31	fixed effect model
	Hyperlipemia	3 [7, 13, 22]	0%	<0.0001	0.76	0.67, 0.86	fixed effect model
	Peripheral vascular disease	2 [12, 13]	59%	0.24	1.21	0.88, 1.66	random effect model
	Hypertension	6 [7, 11, 12, 13, 19, 22]	19%	0.007	1.12	1.03, 1.22	fixed effect model
	Unstable angina	2 [7, 22]	0%	0.63	1.19	0.59, 2.41	fixed effect model
Risk factors for preoperative drug use in patients							
	Anticoagulant drug	2 [7, 13]	0%	0.001	0.77	0.65, 0.90	fixed effect model
	Lipid-regulating drug	2 [7, 13]	0%	0.14	0.88	0.73, 1.05	fixed effect model
	β-blocker	2 [7, 13]	0%	0.09	0.81	0.64, 1.03	fixed effect model
	Renin angiotensin converting enzyme inhibitors	2 [7, 11]	74%	0.23	1.58	0.74, 3.38	random effect model
Risk factors associated with cardiopulmonary bypass							
	Extracorporeal circulation	2 [13, 22]	0%	0.001	1.24	1.09, 1.41	fixed effect model
	Cardiopulmonary bypass time >2 hours	3 [7, 12, 17]	47%	<0.00001	20.26	17.03, 23.48	fixed effect model

### 3.4 Sensitivity Analysis

Sensitivity analysis revealed changes in heterogeneity before and after excluding individual studies. Hyperlipidemia [before excluding a single study [12] (*p* = 0.66, I^2^ = 90%); after excluding the study (*p* < 0.0001, I^2^ = 0%)], Renin angiotensin-converting enzyme inhibitors showed high heterogeneity [before excluding a single study [13] (*p* = 0.58, I^2^ = 89%); after excluding the study (*p* = 0.23, I^2^ = 74%)], and female gender [before excluding a single study [12] (*p* = 0.27, I^2^ = 83%); after excluding the study (*p* < 0.00001, I^2^ = 41%)], there were changes observed. However, risk factors remained stable.

### 3.5 Publication Bias of the Included Studies

The study included a total of 11 published papers [7, 11, 12, 13, 17, 18, 19, 20, 21, 22, 23]. Begg’s test was conducted, and the results indicated a low risk of publication bias among the included studies (Z = –2.65, *p* = 0.0127).

### 3.6 Conduct a Cause Analysis of Publication Bias for the Included Studies

Sources of heterogeneity in the meta-analysis results: Clinical characteristics and disease severity. The severity of the disease in the study subjects, along with the presence of other chronic conditions, may lead to varying outcomes, contributing to heterogeneity. Different study designs can introduce heterogeneity, as each type of study has inherent biases and limitations. Some low-quality studies may lead to publication bias, further contributing to heterogeneity. Methods to reduce sources of heterogeneity include strict inclusion and exclusion criteria: clearly defined, stringent criteria should be applied to ensure consistency in key characteristics of the study population, thereby minimizing variations in outcomes due to sample heterogeneity. Sensitivity and subgroup analysis: When significant heterogeneity is present among the influencing factors, sensitivity and subgroup analyses can be conducted by categorizing and grouping the data. By sequentially excluding studies involving specific influencing factors, the sources of heterogeneity can be identified, thereby reducing heterogeneity and uncovering the effects within specific populations.

## 4. Discussion

Patients in the SHG group were more likely to be over 65 years old than those in the non-SHG group, making age an independent risk factor for SHG after cardiac surgery, which aligns with the findings of Xie Huihui [24]. This study showed a significant association between age and SHG in non-diabetic patients after cardiac surgery, with the severity of SHG increasing with age [25]. Elderly patients often have weakened immune responses and poorer stress responses due to underlying conditions and declining organ function; however, the impact of sex on stress hyperglycemia remains controversial. This study found that female sex is a relevant risk factor for SHG in non-diabetic patients after cardiac surgery, suggesting that older women with poorer cardiac function are more prone to SHG. The release of stress hormones and insulin resistance in response to surgical stress can lead to SHG. Although the mechanism related to female hormones requires further investigation, this study underscores the importance of early prediction and management of SHG in female patients during the perioperative period [26, 27]. Research by ME Ertorer *et al*. [28] on 494 ICU patients with coronary artery disease confirmed sex differences in SHG. Sharma *et al*. [29] also demonstrated a sex-related association with SHG levels. Stress conditions prolong the elevation of hormone levels, contributing to SHG in patients without diabetes post-cardiac surgery. The traumatic nature of surgery, particularly complex procedures like combined valve and coronary artery bypass surgeries, increases the risk of SHG compared to standard cardiac surgeries [30]. Patients with a history of cardiac surgery often experience physiological and metabolic changes, including SHG, consistent with the results of Christos Kourek *et al*. [31]. In patients with a history of heart surgery [32], unique cardiovascular characteristics lead to a more pronounced response to SHG risk factors. Reduced ejection fraction is a significant risk factor for SHG, as declining cardiac function leads to decreased myocardial contractility, a sharp drop in cardiac output, worsening microcirculation, and further exacerbation of myocardial injury, which is consistent with the findings of Stalikas N *et al*. [33]. This meta-analysis also identified hypertension as a risk factor for SHG in patients post-cardiac surgery. Izzo R *et al*. [34] and Conen D [35] found that at admission, systolic blood pressure is an independent risk factor for SHG. Retrospective studies [29] have shown that prehypertensive individuals have higher fasting blood glucose levels than those with ideal blood pressure, indicating that while abnormal blood pressure may not directly cause glucose abnormalities, it does affect blood glucose levels. A study [36] has shown that effective blood pressure control in hypertensive patients helps reduce the incidence of stress-induced hyperglycemia and the development of new-onset diabetes. These mechanisms include hypertension and significant fluctuations in blood pressure, which lead to insulin resistance, sympathetic nervous system activation, Renin-angiotensin-aldosterone system (RAAS) activation, oxidative stress, and inflammation [17], ultimately resulting in stress-induced hyperglycemia [37]. After acute myocardial infarction, the sympathetic nervous system activation increases the levels of glucagon, growth hormones, glucocorticoids, and catecholamines. Research has shown that glucagon is a key factor in gluconeogenesis, promoting hepatic glycogen breakdown, and inducing hyperglycemia. These hormones and cytokines also have complex feedback mechanisms, such as tumor necrosis factor (TNF)-α accelerating gluconeogenesis by stimulating glucagon production [38]. Studies indicate that dyslipidemia affects oxidative stress, impairs β-cell function, and reduces insulin secretion, leading to elevated blood glucose levels [39], with similar findings by Xie Huihui [24] and Abbasi F *et al*. [40]. This study [41] found that preoperative anticoagulant use is closely related to postoperative SHG and is a significant risk factor for SHG, suggesting that patients in a hypercoagulable state or those taking long-term anticoagulants should have their coagulation function closely monitored to ensure normal blood glucose levels preoperatively. Irregular use of warfarin before surgery can lead to stress hyperglycemia and bleeding complications, with irregular warfarin use accounting for 75% of postoperative complications, highlighting its significant impact on SHG in patients without diabetes; extracorporeal circulation time greater than 2 hours [42] was identified as a risk factor for SHG in patients without diabetes. Hormonal secretion of glucagon, glucocorticoids, catecholamines, and growth hormones is affected during surgery, with a decrease in insulin secretion contributing to elevated blood glucose levels. SHG is common in patients undergoing cardiac surgery with extracorporeal circulation time and the likelihood of SHG increases with prolonged extracorporeal circulation time. Extracorporeal circulation increases glucose production, and a prolonged extracorporeal circulation time significantly increases surgical trauma and stress responses, promoting glycogen breakdown and gluconeogenesis. Exogenous glucocorticoids administered during surgery also contribute to perioperative hyperglycemia, making longer extracorporeal circulation times more likely to result in SHG [43, 44]. Low temperatures and high oxygen levels during extracorporeal circulation in cardiac surgery, promote catecholamine release, which inhibits insulin secretion and stimulates glucose production. Low temperatures can directly suppress insulin secretion, potentially causing hypokalemia, which exacerbates insulin resistance [45]. Some researchers believe that high oxygen levels increase hepatic receptor responsiveness to glucagon, thereby elevating blood glucose. Thus, cardiopulmonary bypass and extended bypass duration, low temperatures, and high oxygen states have increasingly pronounced effects on the body. The following criteria can be used to assess high-risk SHG patients after cardiac surgery: Clinical characteristics such as age >65 and gender: Age 65 is considered a threshold, as older patients tend to experience more significant blood glucose fluctuations [24]. Women are at a higher risk of developing stress hyperglycemia after cardiac surgery, possibly because of hormonal changes and differences in metabolic mechanisms [29]. Surgical type and preoperative condition: Combined valve and coronary artery bypass surgery intensifies the postoperative stress response [30]. A reduced ejection fraction indicates weakened heart function, making patients more susceptible to surgical stress, leading to a greater metabolic burden and impaired blood glucose regulation [33]. Patients with a history of cardiac surgery experience increased stress during repeat procedures [31], which increases the risk of SHG. A history of myocardial infarction [38], hyperlipidemia [37], and hypertension [34, 35] may contribute to postoperative metabolic disorders, making blood glucose control challenging. Perioperative anticoagulant use [41], cardiopulmonary bypass and bypass duration >2 hours [43, 44] can also be used as criteria to identify high-risk SHG patients after cardiac surgery, even in patients without diabetes. For patients with identified risk factors, healthcare professionals should implement the following measures during the perioperative period to adjust treatment plans and reduce the incidence of SHG, thereby enhancing the practicality of this study: Preoperative Assessment and Optimization: Conduct a comprehensive evaluation of the patient’s age, gender, type of cardiac surgery, history of cardiac surgery, fasting blood glucose, HbA1c, insulin resistance status, and baseline metabolic level. Preoperative Assessment and Optimization: Conduct a comprehensive evaluation of the patient’s cardiac function before surgery, optimize left ventricular function, and manage heart failure or other cardiac conditions. For patients with impaired cardiac function, preoperative pharmacological treatment may be administered, such as early monitoring and intervention by closely tracking perioperative blood glucose levels, especially in high-risk patients. Effective intraoperative and postoperative insulin management ensures effective insulin management during and after surgery to control blood glucose levels. Preoperative assessment and adjustment of anticoagulant therapy include tailoring the type and dosage of anticoagulant medications based on the patient’s specific circumstances, such as history of thromboembolism or bleeding risk. Use of medications that may cause fluctuations in blood glucose levels. Choosing the appropriate anticoagulants during the perioperative period. Managing Comorbidities: Patients with comorbid conditions such as myocardial infarction, dyslipidemia, and hypertension should be closely monitored. Surgical indicators were adjusted for these patients to ensure that they were within normal ranges before surgery. Extra attention should be paid postoperatively to prevent changes in the patient’s condition and complications. Preoperative blood pressure should be adjusted to normal or within the target range appropriate for surgery. Management During Extracorporeal Circulation: Blood glucose levels were regularly monitored before, during, and after circulation. Continuous infusion of short-acting insulin allows more flexible glucose control during extracorporeal circulation. Additionally, using low-temperature extracorporeal circulation techniques (such as mild or moderate hypothermia) can help reduce the metabolic rate and inflammatory responses; however, the limitations of this study include the variability in the types of studies included and the bias in the selection of risk factors across different studies. To improve the accuracy of the included literature and enhance the reliability of the findings, we applied strict inclusion and exclusion criteria, categorized the studies based on their type, and used different quality assessment tools to reduce bias and heterogeneity in the analysis. In cases where bias and heterogeneity arose from the included studies on risk factors, we employed a stepwise exclusion method to remove studies with high heterogeneity, thereby reducing its impact on the results. We also updated and expanded the literature database, incorporating studies in Chinese, English, non-English languages, and grey literature. In the future, we plan to collaborate with language experts or translation services and design studies in multicenter hospitals specializing in cardiac surgery and endocrinology. This will allow us to handle larger datasets and multilingual literature, ensuring the representativeness of case numbers and the reliability of the data.

## 5. Conclusions

Current evidence suggests that age >65 years, female gender, combined heart valve and coronary artery bypass surgery, ejection fraction<40%, history of heart surgery, myocardial infarction, hyperlipidemia, hypertension, anticoagulant medication, cardiopulmonary bypass time >2 hours and history of cardiopulmonary bypass are key risk factors for postoperative stress hyperglycemia in patients without diabetes who have undergone cardiac surgery. These factors can help identify patients at a high risk of perioperative stress hyperglycemia during cardiac surgery. This evidence provides a basis for healthcare professionals to develop predictive management strategies for perioperative stress hyperglycemia in patients without diabetes period. However, more high-quality studies are required to address the limitations of the current research.

## Availability of Data and Materials

The dataset from this study has been provided as part of the submitted manuscript.

## Supplementary Material

Supplementary material associated with this article can be found, in the online version, at https://doi.org/10.31083/RCM25485.
